# Assessment of laboratory logistics management information system practice for HIV/AIDS and tuberculosis laboratory commodities in selected public health facilities in Addis Ababa, Ethiopia

**DOI:** 10.11604/pamj.2013.15.46.1969

**Published:** 2013-06-08

**Authors:** Adino Desale, Bineyam Taye, Getachew Belay, Alemayehu Nigatu

**Affiliations:** 1 Ethiopian Health and Nutrition Research Institute, Ethopia; 2 College of Health Science, School of Clinical laboratory Science, Addis Ababa University, Addis Ababa, Ethiopia; 3 College of Health Science, School of public health, Addis Ababa University, Addis Ababa, Ethiopia; 4 Management sciences for Health/ Supply Chain Management System, Addis Ababa, Ethiopia

**Keywords:** Logistics management information system, stock outs, laboratory commodities

## Abstract

**Introduction:**

Logistics management information system for health commodities remained poorly implemented in most of developing countries. To assess the status of laboratory logistics management information system for HIV/AIDS and tuberculosis laboratory commodities in public health facilities in Addis Ababa.

**Methods:**

A cross-sectional descriptive study was conducted from September 2010-January 2011 at selected public health facilities. A stratified random sampling method was used to include a total of 43 facilities which, were investigated through quantitative methods using structured questionnaires interviews. Focus group discussion with the designated supply chain managers and key informant interviews were conducted for the qualitative method.

**Results:**

There exists a well-designed logistics system for laboratory commodities with trained pharmacy personnel, distributed standard LMIS formats and established inventory control procedures. However, majority of laboratory professionals were not trained in LMIS. Majority of the facilities (60.5%) were stocked out for at least one ART monitoring and TB laboratory reagents and the highest stock out rate was for chemistry reagents. Expired ART monitoring laboratory commodities were found in 25 (73.5%) of facilities. Fifty percent (50%) of the assessed hospitals and 54% of health centers were currently using stock/bin cards for all HIV/AIDS and TB laboratory commodities in main pharmacy store, among these only 25% and 20.8% of them were updated with accurate information matching with the physical count done at the time of visit for hospitals and health centers respectively.

**Conclusion:**

Even though there exists a well designed laboratory LMIS, keeping quality stock/bin cards and LMIS reports were very low. Key ART monitoring laboratory commodities were stock out at many facilities at the day of visit and during the past six months. Based on findings, training of laboratory personnel's managing laboratory commodities and keeping accurate inventory control procedures were recommended.

## Introduction

Logistics is a branch of management that studies the process of planning, implementing and controlling the efficient, cost effective flow and storage of goods, services from point of origin to point of consumption [1]. Laboratory logistics management information system is the management of laboratory commodities in a systematic and standardized way by collecting, processing and utilizing timely logistics data to inform quantification, procurement, storage and distribution of laboratory commodities [2].

Logistics Management Information System (LMIS) is important for all public health commodity distribution systems. It is especially critical for *Human Immunodeficiency Virus/Acquired Immunodeficiency Syndrome* (HIV/AIDS) commodities that have high value and requires special handling procedures [3].Without LMIS implementation; programs will inevitably waste valuable resources through prolonged and frequent stock outs, overstocks and losses [4]. A well implemented LMIS reduces the likely hood of stock outs and overstocks that can waste scarce resources and lead to product expiration, especially given the short shelf life of HIV test kits [5].

Managing supply chains in support of laboratory services is a formidable challenge, especially in developing countries [2]. Expanding programs for HIV/AIDS, TB and malaria require strong and supportive laboratory services that depend on the availability of the required commodities to perform critical tests, with most tests requiring multiple commodities to be available simultaneously [6].

The Ethiopian laboratory LMIS was weak, consistently being hampered by several systemic challenges that caused frequent stock outs of critical commodities, thus impeding continuous and quality testing for patients [7]. Currently, the country has designed integrated pharmaceutical logistics systems (IPLS) for all public health commodities including essential drugs, family planning, malaria, laboratory services, nutrition, TB-leprosy and HIV/AIDS commodities [8].

Several studies have been conducted to see the status of logistics management information system and supply chain systems for HIV/AIDS and TB laboratory commodities specially for ART drugs and HIV test kits elsewhere, however, there is limited information in Ethiopia in general and particularly in laboratory logistics management information system. Therefore, this study is designed to assess the status of laboratory logistics management information system for HIV/AIDS and TB laboratory commodities at selected public health facilities in Addis Ababa and identify the strengths and weaknesses of the LMIS in order to improve the design and operation of the logistics data collection, analysis and utilization of data for decision of key logistics functions.

## Methods

### Study area

The study was conducted in Addis Ababa which is the capital city of Ethiopia, seat of African Union and Economic Commission. It is located in the geographic centre of the country and covers a landmass of 540 sq. km. It is administratively sub-divided into 10 sub cities and 116 weredas (Lowest level administrative unit in the city) According to central statistical agency 2008 report the city has an estimated population of 2.98 million [9].The city has 45 hospitals, 31 health centers and 551 clinics [10]. The study area was chosen because it is the most accessible area for better implementation of the laboratory logistics system compared to other parts of the country and poor functioning of the system in such area will enable us to see how severe the problem will be in the rural areas of the country.

### Study design and period

A facility based cross-sectional descriptive study was conducted from September 2010-January 2011 on laboratory LMIS practice. Both quantitative and qualitative data collection methods were employed. Interviewing persons responsible for managing laboratory commodities at all levels of the supply chain using structured questionnaire and physical inventory of HIV/AIDS and TB laboratory commodities available at the time of visit was assessed in quantitative part of the study. Focus group discussion with the designated supply chain managers and key informant interviews, using questions adapted from logistics system assessment tool (LSAT), were conducted with central level staffs to understand the challenges facing the logistics systems and to obtain a description of the supply chain system for HIV/AIDS and TB laboratory commodities were done for the qualitative method.

### Source population and study population

All the facilities involved in the supply chain of HIV/AIDS and TB laboratory commodities from central to region and to service delivery points were the source population. A total of 43 health facilities comprising 8 hospitals, 24 health centers, 9 sub-city pharmaceutical supply sustainability case teams, one regional laboratory and one regional health bureau were included in this study using a stratified random sampling method.

### Sample Size Estimation

Sample size was calculated using the minimum sample size calculation formula for cross-sectional study for estimating single proportion assuming 50% prevalence of poorly functioning laboratory LMIS, 5% margin of error and 95% CI due to lack of similar prevalence study in Ethiopia. Finally the correction factor for finite population was applied to include a total of 43 facilities.

### Data collection techniques

A structured questionnaire which is originally developed by DELIVER [11] and locally adapted was used to collect quantitative information from the warehouse and service delivery points. On top of the information collected through interview using the structured questionnaire, physical counts of HIV/AIDS and TB laboratory commodities was done in order to assess data quality by comparing the actual counts with the available records.

The laboratory commodities covered in the assessment were ART monitoring chemistry, hematology and CD4 reagents. In addition RTKs, sample transfer kits (STKs) and AFB testing laboratory reagents were assessed.

The instrument was used to provide information on the indicators like the availability of laboratory commodities for HIV/AIDS and TB diagnostics service on day of visit, stock out frequency and average duration of stock outs, percentage of facilities with personnel trained in logistics, percentage of facilities that had expired commodities, percentage of facilities with stock /bin cards available and accuracy of stock keeping records.

Qualitative method: The quantitative method has provided what information was available regarding the design and operation of the logistics management information system but does not provide how the logistics system functions [12]. The Focus group guide originally developed by DELIVER [13] was locally adapted to be used for this study. Two Focus Group Discussions (FGD) with participants responsible for logistics activities in all the facilities was conducted. The topics that were discussed to identify strengths and weaknesses in the system were flow of HIV/AIDS and TB laboratory commodities in the supply chain, existence and functioning of laboratory LMIS, description of the information flow and how logistics information is used for decision making and capacity of logistics personnel, including training and supervision.

### Statistical analysis

The quantitative data were coded and entered using EPI info 2002 (Centre for Disease Control and Prevention Atlanta, GA) and analyzed using SPSS version 15 software (SPSS INC, Chicago, IL, USA). Descriptive statistics were computed and result was presented using tables and graphs. The qualitative portions of the study (FGD) were analysed using qualitative analysis technique (relistening to the tape recorders several times, transcribing data, categorizing, reducing and finally writing the report by narrating the finding).

### Ethical consideration

The study was first approved by Addis Ababa University (AAU) Institutional Review Board of the Faculty of Medicine (IRB) and research and ethical committee of Addis Ababa Health Bureau before the study was commenced, then a letter informing the facility administrators were written from the school of clinical laboratory science and AARHB. There were a high degree of confidentiality during data collection and no name of any health facility and participating subjects were put in the result instead the aggregate result of the facilities and summary results of focus group discussants were projected.

## Results

A total of 43 public health facilities which are involved in HIV/AIDS and TB laboratory commodity management were investigated in this study, of which 8 (18.6%) were hospitals, 24 (55.8%) health centers, 9 (20.9%) sub-city pharmaceutical supply assuring sustainability case team, 1 (2.3%) Regional Laboratory (RL) and 1 (2.3%) RHB. A total of 75 (65.8%) pharmacy professionals and 39 (34.2%) laboratory professionals were interviewed. (Table 1).

**Table 1:** Characteristics of study participants involved in the assessment of laboratory LMIS for ART monitoring and TB laboratory commodities in Addis Ababa, 2010

Variable	No	%
Facility		
Hospital	8	18.6
Health center	24	55.8
Regional Laboratory	1	2.3
Regional Health Bureau	1	2.3
Sub-city^1^	9	20.9
Total	**43**	**100**
Professionals		
Pharmacy co-ordinators	33	28.9
Pharmacy store managers	33	28.9
Sub-city^2^	9	7.9
Laboratory managers	33	28.9
Laboratory mini-store managers	6	5.4
Total	**114**	**100**

1 Sub-city = sub-city pharmaceutical supply assuring sustainability case team

2 Sub-city = sub-city pharmaceutical supply assuring sustainability case team leader

### Training of professionals in LMIS

From a total of 114 professionals involved in laboratory commodity management, 71 (62.3%) were trained in logistics management information system (integrated pharmaceutical logistics system or Ethiopian laboratory logistics system). of these, 67 (58.8%) were pharmacy professions and 4 (3.5%) were laboratory professionals. (Table 2).

**Table 2:** Training of pharmacy and laboratory professionals in LMIS by facility type

Facility type trained	Pharmacy professionals trained	Laboratory professionals
Hospitals	14(12.3%)	3 (2.6%)
Health centers	42 (36.8%)	0 (0%)
Sub-cities*	9 (7.9%)	___
AARL	____	1(0.9%)
AARHB	2 (1.8)	____
**Total**	67 (58.8%)	4 (3.5%)

* Sub-cities sub-city pharmaceutical supply assuring sustainability case team

__ Respective personnel not available in those facilities

### Stock Availability by Commodity Type

As illustrate in the Table 3 below, six (75%) of facilities had within the established minimum-maximum (min-max) stock levels for creatinine, glucose and cell dyne diluent. Five (62.5%) of facilities had stock on hand within the min-max levels for cell dyne lyze and cell dyne detergents. Six (75%) of facilities had less stock levels than the minimum stock levels for CD4 and bilirubin reagents. Three (37.5%) of facilities had less stock levels than the minimum stock levels for ALP, GPT, BUN, cell dyne lyze and cell dyne detergent reagents.

**Table 3:** Percentage of facilities that had stock on hand below, above and within the minimum/maximum stock levels on the day of visit on Addis Ababa, 2010

Commodities	Facilities with less than the minimum stock level n (%)	Facilities with higher than the maximum stock level n(%)	Facilities within the minimum-maximum stock level n (%)	Median months of stock on hand (25^th^-75^th^ percentile)
CD4 Reagent	6 (75)	0 (0%)	2 (25%)	1.0 (1.0-1.75)
FACS flow	4 (50)	0 (0%)	4 (50%)	1.0 (0.625-1.75)
ALP	3 (37.5%)	2 (25%)	3 (37.5%)	1.5 (1.0-4.25)
GPT	3 (37.5%)	2 (25%)	3 (37.5%)	2.0 (1.0-4.25)
GOT	2 (25%)	2(25%)	4 (50%)	1.5 (1.0-4.5)
Bilirubin (Direct)	6 (75%)	0 (0%)	2 (25%)	0.75 (0.5-1.75)
Bilirubin (Total)	6 (75%)	0 (0%)	2 (25%)	0.75 (0.5-1.00)
BUN	3 (37.5%)	1 (12.5%)	4 (50%)	1.0 (0.625-1.75)
Creatinine	2 (25%)	0 (0%)	6 (75%)	2.0 (1.00-2.00)
Glucose	1 (12.5%)	1 (12.5%)	6 (75%)	1.0 (0.625-2.0)
Cell Dyne Lyze	3 37.5%)	0 (0%)	5 (62.5%)	1.0 (0.625-1.75)
Cell Dyne Detergent	3 37.5%)	0 (0%)	5 (62.5%)	1.0 (0.625-1.75)
Cell Dyne Diluent	2 (25%)	0 (0%)	6 (75%)	1.0 (0.5-1.75)
KHB	15 (45.5%)	2 (6%)	16 (48.5%)	2.0 (1.0-2.0)
Stat-pack	10 (30.3%)	5 (15%)	18 (54.5%)	1.0 (1.0-2.0)
Uni-gold	7 (21.2%)	12 (36.4%)	14 (42.4%)	1.0 (1.0-2.5)
Vacutainer Tube^1^	15 (60%)	6 (24%)	4 (16%)	2.0 (1.0-2.0)
Vacutainer Tube^2^	12 (48%)	5 (20%)	8 (32%)	2.0 (1.0-2.0)
Vacutainer N	15 (60%)	5 (20%)	5 (20%)	1.0 (1.0-2.0)
Carbon fuchsin	10 (29.4%)	8 (23.5%)	16 (47%)	1.0 (1.0-2.0)
Methylene blue	10 (29.4%)	8 (23.5%)	16 (47%)	1.0 (1.0-2.0)
Acid alcohol	15 (44.1%)	6 (17.6%)	13 (338%)	(0.5-2.0)

1 Vacutainer tube (4ml)

2 Vacutainer tube (10ml), vacutainer N- vacutainer needle

*For CD4, Hematology and Chemistry reagents number of facilities that manage them (n = 8)

*For Rapid test kits (n= 33), for sample transfer kits (n = 25), for TB reagent (n = 34)

Two (25%) of facilities had higher stock levels for GPT, GOT and ALP reagents. Two (6%), 5 (15%) and 12 (36.4%) of facilities had higher stock levels for KHB, stat-pack and uni-gold test kits respectively.

### Reported stock outs during the last 6 months

Twenty six (60.5%) of facilities reported that they usually run out of at least one ART monitoring and TB laboratory commodities before resupply. The most frequently stock out ART monitoring commodities were bilirubin reagents, BUN reagents, CD4 reagents and vacutainer tubes with stock out rate 75%, 50%, 50% and 52% respectively. The lowest stock out were uni-gold test kits, stat-pack test kits, carbon fuchsin and Methylene blue reagents with stock out rate 3%, 9%, 11.8% and 11.8% respectively.

### Stock outs at the time of visit

Sixteen facilities (37.2%) had stock outs at the time of visit for at least one laboratory commodity. The highest stock out rate was for bilirubin reagents 4 (50%) followed by vacutainer tubes 10 (40%) and FACS flow reagents 2 (25%) (Table 4).

**Table 4:** Percentage of facilities stock out for laboratory commodities on the day of visit, reported stock outs during the last 6 months, average duration of stock outs and mean number of stock outs in the last 6 months, March-September/2010, Addis Ababa

Commodities	No of Facilities	Facilities stock Out on the day of visit n (%)	Facilities stock out any time in the past months% (n)	Mean # of days (range) of stock outs in the past 6 months	Mean # of times stock outs in the past 6 months
CD4 reagents	8	1 (12.5%)	4 (50%)	6 (1-10)	3
FACS flow	8	2 (25%)	2 (25%)	5 (1-7)	1
ALP	8	1 (12.5%)	3 (37.5%)	40 (10-60)	2
GPT	8	1 (12.5%)	3 (37.5%)	40 (7-60)	2
GOT	8	1 (12.5%)	3 (37.5%)	40 (7-60)	2
Bilirubin (Direct)	8	4 (50%)	75 (6)	73 (15-90)	2
Bilirubin (Total)	8	4 (50%)	75 (6)	73 (15-90)	2
BUN	8	2 (25%)	50 (4)	30 (7-45)	1
Creatinine	8	2 (25%)	25 (2)	21 (5-30)	2
Glucose	8	2 (25%)	25 (2)	15 (5-30)	1
Cell Dyne lyze	8	2 (25%)	37.5 (3)	15 (7-21)	3
CellDyne detergent	8	2 (25%)	25 (2)	15(7-30)	3
Cell Dyne diluent	8	1 (12.5%)	37.5 (3)	15 (3-21)	3
KHB	33	4 (12%)	27.3 (9)	13 (5-20)	3
Stat-pack	33	0 (0%)	9 (3)	3 (5-20)	1
Uni-gold	33	1 (3%)	21 (7)	7 (5-12)	2
Vacutainer tube (4 ml)	25	10 (40%)	52 (13)	5 (3-10)	3
Vacutainer tube (10ml)	25	10 (40%)	52 (13)	5 (2-7)	3
Vacutainer tube needle	25	12 (48%)	40 (10)	6 (5-10)	3
Carbon fuchsin	34	0 (0%)	11.8 (4)	7 (5-15)	1
Methylene blue	34	0 (0%)	11.8 (4)	7 (5-15)	1
Acid alcohol	34	1 (3%)	17.8 (6)	7 (3-10)	2

### Logistics Management Information System

Four (50%) of the assessed hospitals and 13(54%) of health centers were currently using stock/bin cards for all HIV/AIDS and TB laboratory commodities in main pharmacy store. RHB uses both stock cards and bin cards. Similarly regional laboratory starts using bin cards. In hospitals laboratory mini-store 3(37.5%) of them uses stock/bin cards for HIV/AIDS and TB laboratory commodities and 8(37.5%) of health center laboratories starts to use bin cards. Majority of stock/bin cards were not updated with accurate information matching with the physical count done at the time of visit. The total accuracy of stock/bin cards were 38.9.

## Discussion

The ultimate goal of the laboratory logistics system is to ensure laboratory commodity availability at the service delivery points so that clients will receive the necessary laboratory tests. Individuals responsible for managing laboratory commodities need to be trained in timely keeping of records on essential logistics data, analyzing the collected information to make the right decisions and submission of reports to the next higher level.

In this study, it was found that 67 (58.8%) of pharmacy professionals and only 4 (3.5%) laboratory professionals were trained in LMIS. which was comparable with study done by Jabulani N et al. in Zimbabwe [14] where few number of laboratory professionals were trained in LMIS.

The results of the present study showed that, majority of facilities were found under stock for vacutainer tubes, CD4 and bilirubin reagents. In contrast to our finding a study done in Ghana [15] revealed that facilities had adequately stock for CD4 and chemistry reagents. The difference might be due to the fact that the assessment was done in Ghana at the start of ART programme where consumption of these reagents was lower. Our study also showed that, majority of facilities had adequate stock levels for creatinine, glucose, cell dyne diluent, cell dyne lyze and cell dyne detergent reagents, Which was in agreement with study done in Ghana [15].

In the present study, it was found that majority of facilities 26 (60.5%) were stocked out at least one HIV/AIDS and TB laboratory commodities in the past six months. which was comparable with the study done in Tanzania [16].

Our result showed that, most frequent stock out reagents were chemistry reagents (bilirubin and BUN), CD4 reagents in hospitals and vacutainer tubes in health centers, which was comparable with the study done in Rwanda [17]. More over our study showed that from eight facilities that manage haematology reagents, 3 (37.5%) of facilities were stock out for cell dyne reagents in the past 6 months. In contrast to our finding all facilities were stock out for haematology reagents as shown by Pharasi B in Lesotho [18]. The difference might be due to the absence of functional procurement unit for ART monitoring laboratory commodities in Lesotho at that time and our assessment includes only cell dyne haematology reagents.

This study showed that, majority of stock/bin cards were not updated with accurate information matching with the physical count done at the time of visit. The overall accuracy (matching with physical count) of stock/bin cards in all facilities was 38.9%. Similar study in Zimbabwe [14] showed, from 50 (80%) of facilities using stock/bin cards 40 (60%) were updated with accurate information which were much better than our finding. This may be due to the presence of programmed supervision and trained staffs that improved stock/bin card accuracy in Zimbabwe, whereas absence of supportive, programmed supervision and work load as confirmed in FGD were the reasons for low accuracy of stock/bin cards in our case.

Limitation of the study: Lack of similar studies especially in Ethiopia made difficult for comparing results; the sample size was not large enough for some specific indicators

## Conclusion

There is a well-designed logistics system for laboratory commodities and majority of persons responsible in managing commodities in main store (pharmacy store) were trained in LMIS. Majority of laboratory mini-store managers and laboratory managers didn't get LMIS training. A significant number of facilities were stock out for key ART monitoring and TB laboratory commodities at the day of the assessment and during six months before the assessment. Utilization of the logistics recording and stock/bin cards usage were limited to the higher level facilities (Regional health bureaus and hospitals).
